# Waist-to-Height Ratio Is a Stronger Mediator in the Association between DASH Diet and Hypertension: Potential Micro/Macro Nutrients Intake Pathways

**DOI:** 10.3390/nu15092189

**Published:** 2023-05-04

**Authors:** Min Yuan, Qi Li, Can Yang, Liping Zhi, Weiwei Zhuang, Xu Steven Xu, Fangbiao Tao

**Affiliations:** 1 School of Public Health Administration, Anhui Medical University, Hefei 230032, China; 2 School of Management, University of Science and Technology of China, Hefei 230026, China; 3 Clinical Pharmacology and Quantitative Science, Genmab Inc., Princeton, NJ 08536, USA; 4 School of Public Health, Anhui Medical University, Hefei 230032, China; 5 MOE Key Laboratory of Population Health across Life Cycle, Hefei 230032, China

**Keywords:** anthropometric measurements, DASH score, hypertension, micro/macronutrients, mediation analysis

## Abstract

Several studies have demonstrated that adhering to the Dietary Approaches to Stop Hypertension (DASH) diet may result in decreased blood pressure levels and hypertension risk. This may be an effect of a reduction in central obesity. In the current study, we explored the mediation role of multiple anthropometric measurements in association with DASH score and hypertension risk, and we investigated potential common micro/macro nutrients that react with the obesity-reduction mechanism. Our study used data from the National Health and Nutrition Examination Survey (NHANES). Important demographic variables, such as gender, race, age, marital status, education attainment, poverty income ratio, and lifestyle habits such as smoking, alcohol drinking, and physical activity were collected. Various anthropometric measurements, including weight, waist circumference, body mass index (BMI), and waist-to-height ratio (WHtR) were also obtained from the official website. The nutrient intake of 8224 adults was quantified through a combination of interviews and laboratory tests. We conducted stepwise regression to filter the most important anthropometric measurements and performed a multiple mediation analysis to test whether the selected anthropometric measurements had mediation effects on the total effect of the DASH diet on hypertension. Random forest models were conducted to identify nutrient subsets associated with the DASH score and anthropometric measurements. Finally, associations between common nutrients and DASH score, anthropometric measurements, and risk of hypertension were respectively evaluated by a logistic regression model adjusting for possible confounders. Our study revealed that BMI and WHtR acted as full mediators between DASH score and high blood pressure levels. Together, they accounted for more than 45% of the variation in hypertension. Interestingly, WHtR was found to be the strongest mediator, explaining approximate 80% of the mediating effect. Furthermore, we identified a group of three commonly consumed nutrients (sodium, potassium, and octadecatrienoic acid) that had opposing effects on DASH score and anthropometric measurements. These nutrients were also found to be associated with hypertension in the same way as BMI and WHtR in univariate regression models. The most important among these nutrients was sodium, which was negatively correlated with the DASH score (β = −0.53, 95% CI = −0.56~−0.50, *p* < 0.001) and had a positive association with BMI (β = 0.04, 95% CI = 0.01~0.07, *p* = 0.02), WHtR (β = 0.06, 95% CI = 0.03~0.09, *p* < 0.001), and hypertension (OR = 1.09, 95% CI = 1.01~1.19, *p* = 0.037). Our investigation revealed that the WHtR exerts a greater mediating effect than BMI on the correlation between the DASH diet and hypertension. Notably, we identified a plausible nutrient intake pathway involving sodium, potassium, and octadecatrienoic acid. Our findings suggested that lifestyle modifications that emphasize the reduction of central obesity and the attainment of a well-balanced micro/macro nutrient profile, such as the DASH diet, could potentially be efficacious in managing hypertension.

## 1. Introduction

Hypertension is a leading cause of cardiovascular mortality and morbidity worldwide. World Health Organization (WHO) reported that nearly 1.1 billion people around the world suffer from hypertension, with one in four men and one in five women affected [1,2]. Its etiology is multifaceted, and researchers suggest that one’s dietary pattern could be a contributing factor to the onset of hypertension [3,4]. The Dietary Approaches to Stop Hypertension (DASH) diet is designed as a hypertension-preventing dietary pattern that advocates the intake of fruits, vegetables, whole grains, low-fat dairy products, and lean meats [5].

The DASH trial was a randomized controlled trial that investigated the effect of DASH dietary pattern on blood pressure levels in adults with prehypertension or stage 1 hypertension [5]. The study showed that the DASH diet was most effective at reducing blood pressure levels. Additionally, the study revealed that people without hypertension could also benefit from the DASH diet as it may help prevent hypertension. The DASH score is an index designed to capture the dietary intake features of the DASH diet and is a marker of adherence to the DASH diet. Research has shown that adhering to the DASH diet can significantly lower blood pressure levels and mitigate the likelihood of hypertension, especially for individuals with high blood pressure, rendering it a potent nutritional approach for the prevention and control of hypertension [6].

Obesity is another public concern due to its impact on health, the economy, the environment, and societal well-being. The WHO reported that worldwide obesity has nearly tripled since 1975 in both the adult and child population. Obesity is a substantial risk factor for the development of hypertension and cardiovascular disease. Studies have revealed that the incidence of obesity is rising in correlation with the occurrence of hypertension [7,8,9]. Overweight or obese individuals constitute approximately two-thirds of the hypertensive patients [10,11]. Being overweight or obese causes metabolic abnormalities that increase the risk of high blood pressure. Anthropometric measurements such as body mass index (BMI) and waist-to-height ratio (WHtR) are commonly used to assess obesity and serve as effective surrogates for metabolic changes that prevent hypertension [7,12,13]. Numerous investigations have demonstrated a steady, affirmative association between elevated BMI/WHtR and an increased probability of developing high blood pressure, even after controlling for variables such as age, gender, and smoking habits [14,15]. Although the DASH diet has been proven effective in reducing blood pressure, the underlying mechanisms responsible for its beneficial effects remain unclear. Certain research has indicated that the DASH diet’s capacity to decrease weight could be a factor in its effectiveness for reducing blood pressure levels [16].

Our current study explored the relationship between the adherence to the DASH diet and hypertension, and it evaluated the potential for anthropometric measurements to be a surrogate for metabolic markers in a free-living setting based on a pubic dataset from NHANES, which is an observational study that assesses the health and nutrition status of both adults and children in the United States. In addition, we also sought to explore how particular micro/macro nutrients in the diet may assist in optimizing obesity-related anthropometric measurements in the US population.

## 2. Materials and Methods

### 2.1. Study Population

We obtained data from the official National Health and Nutrition Examination Survey (NHANES) website (http://www.cdc.gov/nchs/nhanes.htm (accessed on 20 October 2022)). NHANES is a survey that assesses the health and nutrition status of both adults and children in the United States. The survey comprises an interview section and an examination section. By utilizing the 24 h dietary recall method, comprehensive information regarding the specific types and quantities of foods and beverages that are consumed by individuals in America were gathered. To convert dietary nutrients into corresponding food pattern components, we used the Food Patterns Equivalents Database (FPED). Then, DASH score was established by its own scoring system. Our analysis incorporated important demographic covariates, various anthropometric measurements, and multiple micro/macronutrients for underlying nutrient pathway analysis. Participants that met the eligibility criteria of the NHANES and did not have missing values were included in our analysis. The NHANES database is a publicly accessible resource that has been granted approval by the National Center for Health Statistics’ Institutional Review Board. Furthermore, all study participants provided written informed consent prior to taking part in the research.

### 2.2. Demographic, DASH Diet, Anthropometric Measurements and Nutrients Assessment

Several covariates were collected from the NHANES database, including age, gender (male/female), race/ethnicity (non-Hispanic white, non-Hispanic black, Hispanic and others), years of education (divided into four levels: less than high school, high school, college graduate or higher), marital status (living alone or not), poverty income ratio (PIR), tobacco consumption (never smoked, former smoker, current smoker), alcohol drinking (less than 4 drinks per day or more than 4 drinks per day), sodium intake and physical activity.

Physical activity levels of the participants were assessed utilizing information from the questionnaire section of the NHANES public database. The questionnaire included details on the frequency and duration of both moderate- and vigorous-intensity activities that were performed continuously for at least 10 min per week. The weekly amount of physical activity was computed by multiplying the frequency and duration of each activity type. Then, the total weekly physical activity was determined using the moderate-intensity equivalent minutes formula [17]. In line with established aerobic guidelines [17], participants were classified as inactive (<10 min per week), insufficiently active (10–149 min per week), sufficiently active (150–299 min per week), or highly active (≥300 min per week) based on their moderate-intensity equivalent minutes.

Four distinct anthropometric measurements were included in our assessment. In NHANES, a stadiometer was used to measure height, which is a device comprising a vertical ruler attached to a base. Weight was measured using a calibrated digital scale. Waist circumference was measured using a flexible tape wrapped around the bare abdomen at the level of the iliac crest. NNHANES also reported individual BMI by dividing the weight by the height (kg/m²). Additionally, the waist-to-height ratio (WHtR) was determined by dividing the waist circumference by the standing height of the participant.

The DASH score, proposed by Fung et al. [18], is a scoring system that assesses eight food groups and components that are relevant to the DASH dietary pattern. The scoring system places a strong emphasis on the consumption of fruits, whole grains, low-fat dairy products, vegetables, lean meats, and fish, while simultaneously limiting the intake of sodium, saturated and trans-fats, and added sugars. Details on how to calculate the DASH score are provided elsewhere [18,19].

To collect data on macro and micronutrient intake, NHANES used a combination of dietary recall interviews, dietary behavior questionnaires, and biochemical measurements. There were sixty nutrients quantified. Macronutrients included protein, lipids, and carbohydrates. Fat included 8 saturated fatty acids and 11 unsaturated fatty acids. Micronutrients included 23 types of vitamins and 9 types of minerals. We also included dietary fiber and water in the analysis. Detailed information for specific nutrient variables were provided in Supplementary materials (Table S1).

### 2.3. Hypertension

In the NHANES survey, a trained nurse recorded participants’ blood pressure following the standard protocol. Participants were requested to sit calmly for five minutes before four consecutive blood pressure readings were taken. Participants who had completed four blood pressure readings discarded the first one. The mean value of the subsequent three readings was computed to determine blood pressure. Blood pressures were averaged directly if less than four blood pressure readings were available. Hypertension was determined according to the American College of Cardiology and American Heart Association’s guidelines (version 2017), which stipulate a systolic blood pressure (SBP) of 130 mmHg or above, or a diastolic blood pressure (DBP) of 80 mmHg or above [20].

### 2.4. Statistical Analysis

**Figure 1:**
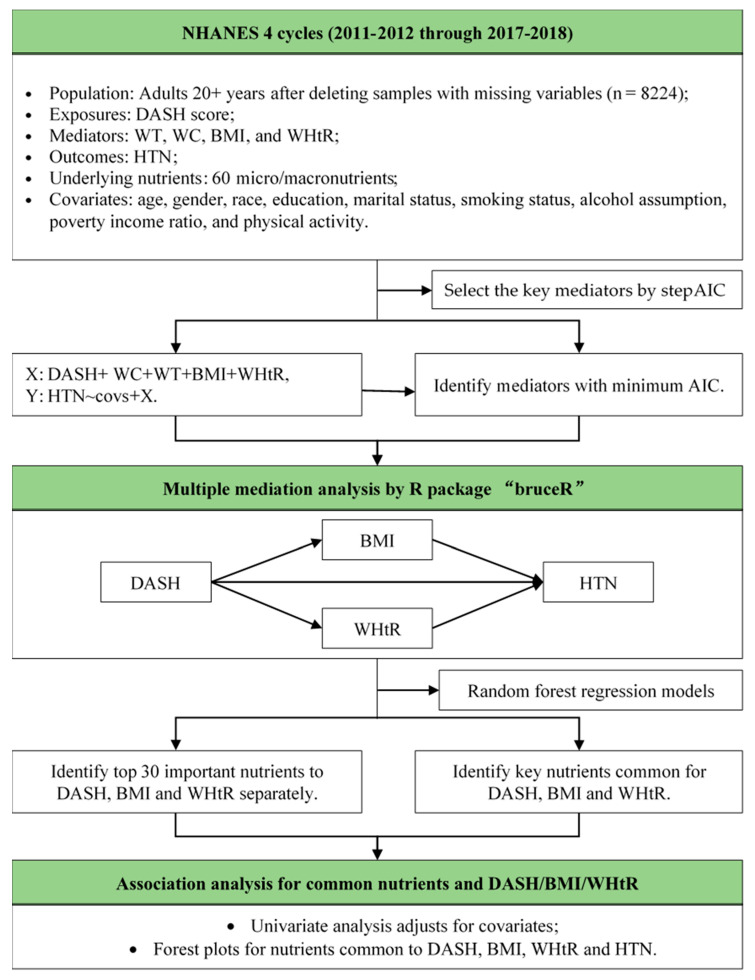
Schematic diagram of study design and analysis. WT: weight; WC: waist; BMI: body mass index; WHtR: waist-to-height ratio; HTN: hypertension status; DASH: Dietary Approaches to Stop Hypertension.

The R software version 4.0.2 was used to conduct statistical analysis. A flowchart of the data processing and statistical analysis procedure is presented in Figure 1. We characterized the variable distribution according to the American Heart Association’s hypertension stage classification. Participants were classified into three categories, namely normal blood pressure, hypertension-stage 1, and hypertension-stage 2 or above. As there is no consensus in the literature on the threshold for DASH scores to define a good and a poor DASH diet, we adopted the cut-off point of 30, which was recommended by Fung et al. [18]. To analyze continuous variables, we computed the median and interquartile range for every HTN stage. On the other hand, for categorical variables, we provided subgroup counts and proportions for each HTN stage. Pearson’s Chi-squared test was used to test independence between categorical variables and hypertension stages, and Kruskal–Wallis rank sum test was used to test differences among different hypertension stages for continuous variables. Corresponding p values were reported, and statistical significance was defined as *p* < 0.05. Centering and scaling procedures were applied to continuous variables for further analysis. Due to a high correlation between the anthropometric measurements, stepwise regression was employed to identify the most significant ones. A multiple mediation analysis was then carried out to investigate the mediating effects of the selected anthropometric measurements in the association between the DASH diet and hypertension.

To further explore the relationship between DASH score and obesity (as measured by anthropometric indicators), we utilized random forest regression models to investigate the potential micronutrients and macronutrients associated with these factors. Variable importance was determined by the average decrease in node impurities resulting from the splitting of that variable. Of the 60 nutrients investigated, we identified the top 30 most impactful nutrients for DASH and anthropometric measurements, respectively. Subsequently, we identified a panel of nutrients that were common across all three traits. To further elucidate the effects of these nutrients on DASH, anthropometric measurements, and hypertension, we conducted univariate analysis while controlling for age, gender, race, marital status, smoking habits, alcohol consumption, and physical activity.

## 3. Results

Table 1 presented the demographic characteristics of the study participants based on their hypertension status. Generally, the study population comprised 8224 participants aged 20–80 years old, 4455 men (54.2%) and 3769 women (45.8%). More than half of the participants had achieved high school (54.2%), and about 2450 (29.8%) participants had obtained a university degree. Hispanic, non-Hispanic white and non-Hispanic black accounted for 22.4%, 21.0% and 56.6% of the study population. In addition, 40.9% participants lived alone. It appears that participants that had good adherence to the DASH diet were more likely to have normal blood pressure compared to those who had a poor DASH score (*p* = 0.027). In addition, higher a BMI was observed in participants with hypertension compared to those with normal blood pressure. This study aimed to examine the correlation between following a DASH diet pattern and hypertension in a sample of 8224 adult from the NHANES, out of which 2852 were diagnosed with hypertension.

We performed logistic regression analysis to explore the association between DASH score and hypertension (HTN) while controlling for potential confounding variables with and without anthropometric measurements. Table S2 shows that the odds ratio (OR) increased from 0.8851 to 0.9323, and the corresponding *p*-values changed from highly significant (*p* = 7.15 × 10^−7^) to non-significant (*p* = 0.136), implying that the relationship between DASH score and HTN may be partially mediated by obesity. To address the high collinearity between anthropometric measurements, we conducted stepwise regression to identify independent variables and eliminate possible collinearity. BMI and WHtR were retained due to their lower Akaike Information Criterion (AIC) values.

Moreover, we performed a formal multiple mediation analysis to evaluate the mediating role of BMI and WHtR in the relationship between DASH score and HTN, as shown in Table 2. Our findings revealed that BMI and WHtR partially mediated the inverse correlation between DASH score and HTN, with a significant indirect effect (beta: −0.013 and *p* = 0.025). Taken together, BMI and WHtR accounted for 45% of the indirect effects, with WHtR being the primary contributor (36%). In order to verify the robustness of our results, we conducted a sensitivity analysis by excluding the lifestyle habit covariates (SMQ, ALQ, and physical activity), and the mediation analysis still demonstrated that BMI and WHtR explained 41% of the indirect effect.

In the following step, we used a machine learning methodology to uncover a shared pathway underlying nutrient intake across the DASH score, BMI, and WHtR. To accomplish this, we applied random regression models to the 60 nutrients individually for each of the three traits. Next, we sorted the 60 nutrients in descending order according to their node purities and selected the top 30 nutrients for each trait. Among these nutrients, 18 were found to be shared across DASH score, BMI, and WHtR, and we refer to them collectively as the “panel of nutrients”. These 18 nutrients jointly accounted for 68.88%, 8.90%, and 16.44% of the variance observed in DASH score, BMI, and WHtR, respectively, after adjusting for possible confounding factors.

**Figure 2:**
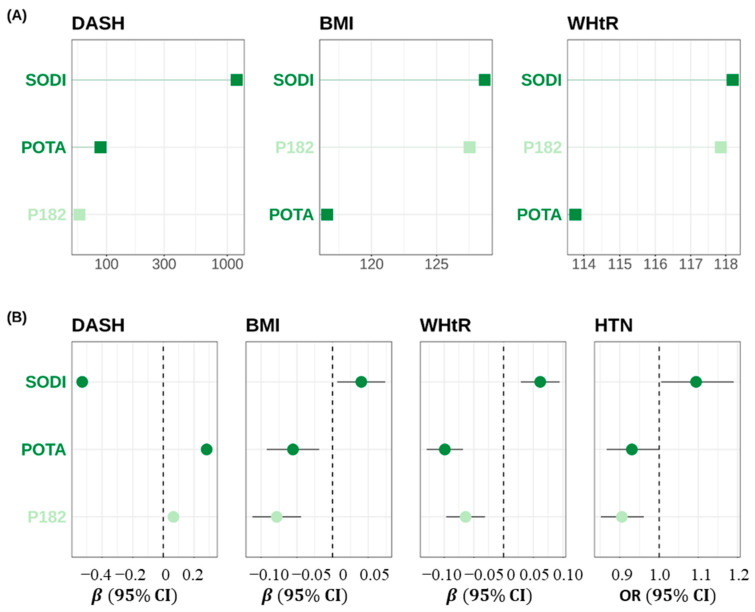
(**A**) Increase in node purity for the three common nutrients for DASH, BMI and WHtR based on random forest models. (**B**) Effects by regressing DASH, WHtR, HTN on the panel of nutrients identified above. Betas were reported for DASH and WHtR and for odds ratio for HTN. CI: confidence interval.

Further, we conducted multiple regression analyses for DASH score, BMI, and WHtR and 18 common nutrients, leading to the identification of three significant nutrients: sodium (SODI), unsaturated fatty acids (octadecatrienoic acid, P182), and potassium (POTA). Based on our research, it was found that SODI had the most significant impact on DASH score, BMI, and WHtR, consistent with existing studies. Additionally, the levels of SODI, P182, and POTA were significantly associated with DASH, BMI, and WHtR, with *p*-values ranging from 0.003 to <0.001. In terms of correlations, SODI displayed a negative association with DASH but a positive association with BMI/WHtR. On the other hand, the POTA and P182 exhibited a positive correlation with DASH and a negative correlation with BMI/WHtR. We also found that covariate-adjusted SODI was associated with an increased risk of hypertension, with OR values of 1.0943 (95% CI = 1.0053~1.1905; *p* = 0.037). In contrast, POTA and P182 were associated with a lower risk of hypertension, with OR values of 0.9306 (95% CI = 0.8665~0.9986, *p* = 0.047) and 0.9051 (95% CI = 0.8518~0.9610, *p* = 0.001) for POTA and P182, respectively. Results were presented in Figure 2. These results remained consistent even after further adjusting for BMI and DASH score, although the effects were attenuated.

## 4. Discussion

The prevalence of hypertension characterized by elevated blood pressure has increased in recent years. The relationship between DASH score and hypertension has been well established, but the mechanisms underlying this relationship are not still unclear. Based on the large population in the NHANES, we revealed that the association between DASH score and hypertension could potentially be mediated by several anthropometric measurements, which are known surrogates of metabolic conditions. Using stepwise model selection method, BMI and WHtR were identified as the crucial ones among four anthropometric measurements (WT, WAIST, BMI and WHtR). BMI and WHtR are two important anthropometric measurements that provide information about body composition. BMI estimates the overall body fat, while WHtR provides information about the distribution of body fat. Several studies have documented that the risk for hypertension increased with the presence of central obesity, as indicated by an elevated WHtR, even in individuals with a normal BMI. Therefore, multiple mediation analyses by combining BMI and WHtR may provide more indirect information while linking DASH to hypertension.

The DASH diet has been proposed to have a positive impact on obesity, which may explain how it helps to lower the risk of hypertension. A recent study based on the cross-sectional TwinsUK cohort showed that BMI mediated this relationship [21]. The current study showed that the mediation portion explained by WHtR was four times greater than that explained by BMI. This finding suggests that WHtR may serve as a more significant mediator than BMI in the correlation between DASH score and hypertension within the US population.

We further investigated the potential micro and macronutrient intake pathways. Using random forest models, we identified a subset of 18 common nutrients that relate to both the DASH score and BMI/WHtR, but with opposite effects. We refined the 18 nutrients to three significant nutrients by multiple regression models while adjusting for demographic and life style variables. The results are presented in Figure 2B. Not surprisingly, sodium was identified as the most important contributor to both BMI/WHtR and DASH score. Additionally, we observed a positive correlation between hypertension and sodium, which was negatively linked to DASH score but positively linked to BMI and WHtR. These results revealed that the excessive consumption of sodium might elevate blood pressure, which is consistent with previous results [22,23,24].

In the present study, we also identified potassium as a possible micronutrient in the pathway relating DASH score and the anthropometric measurements. We found that potassium was positively associated with adherence to the DASH diet. Conversely, we observed a negative correlation between potassium intake and BMI, as well as WHtR, which were both negatively correlated with hypertension. Some researchers have suggested that the opposite effect of sodium and potassium might be explained by the fact that potassium works by counteracting the effects of sodium [25,26,27,28,29,30]. Therefore, implementing dietary modifications to reduce sodium intake and balance potassium intake, such as adopting the DASH diet, can be an effective approach for preventing and managing hypertension. Our findings were further supported by the additional adjustments of both BMI and DASH score, which resulted in consistent results, albeit with weakened effects, thus corroborating the role of BMI and WHtR as potential mediators in the DASH diet–hypertension relationship.

Octadecatrienoic acid, also known as omega-3 fatty acid, is a type of polyunsaturated fat that has been associated with a wide range of health benefits, especially for cardiovascular risk reduction. In the current study, we identified octadecatrienoic acid as the third most important micronutrient. A positive correlation between octadecatrienoic acid and adherence to the DASH diet was discovered, whereas negative associations were found between octadecatrienoic acid and measurements of body obesity and hypertension risk. These results suggest that foods rich in octadecatrienoic acid may have beneficial effects on reducing BMI/WHtR, leading to a lower risk of hypertension.

It should be noted that the initial testing of the blood-pressure-lowering effects of the DASH trial was conducted under stable weight, constant sodium intake levels, and limited alcohol consumption condition; therefore, we examined the association between DASH score and hypertension while adjusting for BMI, sodium, and alcohol consumption as covariates in the model to eliminate possible confounding effects. The association was still statistically significant, indicating that our results in free living situations were still valid. In addition, we included energy-based sodium intake as one of the important confounders when examining the relationship between DASH score and blood pressure, as the DASH diet was tested with a constant and typical sodium level. Results showed that our results were robust with respect to the potential impact of sodium intake.

## 5. Conclusions

We investigated the role of several anthropometric measurements that are known surrogates for metabolic markers in mediating the association between DASH score and blood pressure levels and hypertension. The WHtR was found to be a more effective mediator than body mass index (BMI) as its mediating ratio was four times that of BMI. Thus, reducing WHtR may be a key target in the prevention and control of hypertension.

In addition, this paper explored the relationship between the intake of various nutrients and DASH score and WHtR. The results suggested that sodium, potassium, and omega-3 fatty acids are the most important nutrients related to WHtR. To reduce the risk of hypertension, it is recommended to restrict sodium intake, balance potassium intake, and consume foods rich in omega-3 fatty acids. These interventions can indirectly regulate metabolism and ultimately help manage blood pressure and prevent hypertension.

## Figures and Tables

**Table 1:** Participant characteristics according to hypertension stages defined by AHA.

	Hypertension Stages			
	Normal (5234)	HTN Stage 1 (1681)	HTN Stage 2 (1309)	*p*
**Categorical Variables, Number (%)**				
**Gender**				<0.001
Male	2631 (50)	1043 (62)	781 (60)	
Female	2603 (50)	638 (38)	528 (40)	
**Education**				<0.001
<High school	764 (15)	283 (17)	272 (21)	
High school	2800 (53)	926 (55)	729 (56)	
College and above	1670 (32)	472 (28)	308 (24)	
**Race/Ethnic**				<0.001
Hispanic	1227 (23)	363 (22)	251 (19)	
None-Hispanic White	2323 (44)	730 (43)	526 (40)	
None-Hispanic Black	944 (18)	380 (23)	400 (31)	
Other	740 (14)	208 (12)	132 (10)	
**Marital Status**				0.4
Married	3074 (59)	1017 (60)	764 (58)	
Live alone	2160 (41)	664 (40)	545 (42)	
**Smoking Status**				<0.001
Never	2895 (55)	828 (49)	601 (46)	
Former	1136 (22)	456 (27)	397 (30)	
Current	1203 (23)	397 (24)	311 (24)	
**Alcohol Assumption**				<0.001
≤4 drinks/day	1823 (35)	694 (41)	561 (43)	
>4 drinks/day	3411 (65)	987 (59)	748 (57)	
**Physical Activity**				0.2
<10 min/week	2114 (40)	680 (40)	580 (44)	
10 min~149 min/week	1086 (21)	353 (21)	253 (19)	
150 min~299 min/week	416 (7.9)	134 (8.0)	111 (8.5)	
≥300 min/week	1618 (31)	514 (31)	365 (28)	
**DASH score**				0.027
Good (DASH score >= 30)	1205 (23)	335 (20)	285 (22)	
Poor (DASH score < 30)	4029 (77)	1346 (80)	1024 (78)	
**Continuous Variable,** **Median (IQR)**				
**Age, year**	40 (29, 54)	50 (37, 62)	59 (47, 69)	<0.001
**Poverty Income Ratio (PIR)**	2.46 (1.21, 4.63)	2.55 (1.23, 4.67)	2.14 (1.13, 3.92)	<0.001
**Systolic blood pressure (SBP)**	114 (107, 120)	131 (125, 135)	147 (142, 157)	<0.001
**Diastolic blood pressure (DBP)**	68 (62, 73)	80 (71, 84)	82 (70, 91)	<0.001
**Body Mass Index (BMI)**	27 (24, 32)	29 (25, 34)	30 (25, 34)	<0.001
**Waist-to-height Ratio (WHtR)**	0.13 (0.11, 0.15)	0.14 (0.12, 0.16)	0.15 (0.13, 0.17)	<0.001
**Weight (WT)**	77 (66, 91)	83 (72, 99)	85 (69, 100)	<0.001
**WAIST**	21.3 (18.7, 24.5)	23.5 (20.8, 26.6)	23.9 (21.0, 27.5)	<0.001

Note: Pearson’s Chi-squared test for categorical variable and Kruskal–Wallis rank sum test for continuous variables. A *p*-value less than 0.05 was considered statistically significant.

**Table 2:** Mediation Model: DASH score on risk of hypertension (HTN) (*n* = 8224).

	DASH Effect on Mediator (Path a: X->M)				Multiple Mediator Model (Path b: M->HTN)			
		95% CI				95% CI		
Mediator	Beta	Lower	Upper	*p*	Beta	Lower	Upper	*p*
**BMI**	−0.116	−0.139	−0.093	<0.001	0.018	−0.008	0.045	0.178
**WHtR**	−0.141	−0.163	−0.118	<0.001	0.058	0.031	0.086	<0.001
	**Indirect effect** **(Path ab: DASH->M->HTN)**			**% Mediated** **(ab/c)**	**Total effect** **(Path c: DASH->HTN)**			
		**95% CI**		**Multiple** **mediation model**		**95% CI**		
**Mediator**	**Beta**	**Lower**	**Upper**		**Beta**	**Lower**	**Upper**	***p***
**BMI**	−0.002	−0.006	0.001	9.09%	−0.023	−0.032	−0.012	<0.001
**WHtR**	−0.008	−0.012	−0.004	36.36%	**Direct effect**			
					**(Path c’: DASH->M->HTN)**			
						**95% CI**		
**Total**	−0.010	−0.018	−0.003	45.45%	**Beta**	**Lower**	**Upper**	***p***
					−0.013	−0.024	−0.002	0.025

Note: Adjust for AGE, GENDER, RACE, MARRY, EDUC, PIR, SMQ, ALQ, sodium and PA. CI = Confidence Interval; X = DASH; M = Body Mass Index (BMI) or Waist-to-Height Ratio (WHtR).

## Data Availability

The data that support the findings of this study are publicly available at the official website of NHANES (http://www.cdc.gov/nchs/nhanes.htm (accessed on 20 October 2022)).

## Supplementary Materials

The following supporting information can be downloaded at: https://www.mdpi.com/article/10.3390/nu15092189/s1, Table S1: Nutrients used in current study; Table S2: Association results of regressing DASH/BMI/WHtR/HTN on nutrients.
