# Supplementary material for: Waist-to-Height Ratio Is a Stronger Mediator in the Association between DASH Diet and Hypertension: Potential Micro/Macro Nutrients Intake Pathways
Source: Nutrients. 2023 May 4;15(9):2189. doi: 10.3390/nu15092189 (PMC10180957; doi:10.3390/nu15092189)
Supplement: Supplementary file 1 [file nutrients-15-02189-s001.zip › nutrients-2336125-supplementary.pdf]

nutrients

MDPI

Supplementary Materials

Waist-to-Height Ratio is a Stronger Mediator in the Association between DASH Diet and Hypertension: Potential Micro/Macro Nutrients Intake Pathways

Min Yuan [1,*], Qi Li [1], Can Yang [1], Liping Zhi [1], Weiwei Zhuang [2], Xu Steven Xu [3] and Fangbiao Tao [4,5,*]

Table S1. Nutrients used in current study.

Category (count)	NHANES Label	Variable Description	Unit
Carbohydrate (1)	DR1TCARB	Carbohydrate	gm
Protein (1)	DR1TPROT	Protein	gm
Lipids (5)	DR1TTFAT	Total fat	gm
	DR1TSFAT	Total saturated fatty acids	gm
	DR1TMFAT	Total monounsaturated fatty acids	gm
	DR1TPFAT	Total polyunsaturated fatty acids	gm
	DR1TCHOL	Cholesterol	mg
Saturated fatty acid (8)	DR1TS040	SFA 4:0(Butanoic)	gm
	DR1TS060	SFA 6:0(Hexanoic)	gm
	DR1TS080	SFA 8:0(Octanoic)	gm
	DR1TS100	SFA 10:0(Decanoic)	gm
	DR1TS120	SFA 12:0(Dodecanoic)	gm
	DR1TS140	SFA 14:0(Tetradecanoic)	gm
	DR1TS160	SFA 16:0(Hexadecanoic)	gm
	DR1TS180	SFA 18:0(Octadecanoic)	gm
Monounsaturated fatty acids (4)	DR1TM161	MFA 16:1(Hexadecenoic)	gm
	DR1TM181	MFA 18:1(Octadecenoic)	gm
	DR1TM201	MFA 20:1(Eicosenoic)	gm
	DR1TM221	MFA 22:1(Docosenoic)	gm
Polyunsaturated fatty acids (7)	DR1TP182	PFA 18:2(Octadecadienoic)	gm
	DR1TP183	PFA 18:3(Octadecatrienoic)	gm
	DR1TP184	PFA 18:4(Octadecatetraenoic)	gm
	DR1TP204	PFA 20:4(Eicosatetraenoic)	gm
	DR1TP205	PFA 20:5(Eicosapentaenoic)	gm
	DR1TP225	PFA 22:5(Docosapentaenoic)	gm
	DR1TP226	PFA 22:6(Docosahexaenoic)	gm
Vitamin (23)	DR1TATOC	Vitamin E as alpha-tocopherol	mg
	DR1TATOA	Added alpha-tocopherol (Vitamin E)	mg
	DR1TRET	Retinol	mcg
	DR1TVARA	Vitamin A, RAE	mcg
	DR1TACAR	Alpha-carotene	mcg
	DR1TBCAR	Beta-carotene	mcg
	DR1TCRYP	Beta-cryptoxanthin	mcg
	DR1TLYCO	Lycopene	mcg
	DR1TLZ	Lutein + zeaxanthin	mcg
	DR1TVB1	Thiamin (Vitamin B1)	mg
	DR1TVB2	Riboflavin (Vitamin B2)	mg
	DR1TNIAC	Niacin	mg

	DR1TVB6	Vitamin B6	mg
	DR1TFOLA	Total folate	mcg
	DR1TFA	Folic acid	mcg
	DR1TFF	Food folate	mcg
	DR1TFDFE	Folate, DFE	mcg
	DR1TCHL	Total choline	mg
	DR1TVB12	Vitamin B12	mcg
	DR1TB12A	Added vitamin B12	mcg
	DR1TVC	Vitamin C	mg
	DR1TVD	Vitamin D (D2 + D3)	mcg
	DR1TVK	Vitamin K	mcg
Minerals (9)	DR1TCALC	Calcium	mg
	DR1TPHOS	Phosphorus	mg
	DR1TMAGN	Magnesium	mg
	DR1TIRON	Iron	mg
	DR1TZINC	Zinc	mg
	DR1TCOPP	Copper	mg
	DR1TSODI	Sodium	mg
	DR1TPOTA	Potassium	mg
	DR1TSELE	Selenium	mcg
Dietary fiber (1)	DR1TFIBE	Dietary fiber	gm
Water (1)	DR1TMOIS	Moisture	gm

Table S2. Association results of regressing DASH/BMI/WHtR/HTN on nutrients.

**Endpoint**	**Nutrients**	**Category**	**Est**	**95% CI**		***p***
				**lower**	**upper**	
DASH	SODI	Mineral	−0.5298	−0.5564	−0.5031	<0.001
	POTA	Mineral	0.2831	0.2545	0.3116	<0.001
	P182	Nonsaturated	0.0663	0.0358	0.0968	<0.001
BMI	SODI	Mineral	0.0403	0.0067	0.0739	0.0189
	POTA	Mineral	−0.0555	−0.0921	−0.0188	0.0030
	P182	Nonsaturated	−0.0782	−0.1123	−0.0441	<0.001
WHtR	SODI	Mineral	0.0618	0.0292	0.0943	0.0002
	POTA	Mineral	−0.0987	−0.1293	−0.0681	<0.001
	P182	Nonsaturated	−0.0638	−0.0964	−0.0311	0.0001
HTN	SODI	Mineral	1.0943	1.0053	1.1905	0.0366
	POTA	Mineral	0.9306	0.8665	0.9986	0.0470
	P182	Nonsaturated	0.9051	0.8518	0.9610	0.0012

*Est: regression coefficient for DASH, BMI and WHtR; Odds Ratio for HTN.
